# Toxicity of Nanoparticles on the Reproductive System in Animal Models: A Review

**DOI:** 10.3389/fphar.2017.00606

**Published:** 2017-09-05

**Authors:** Rahim Dad Brohi, Li Wang, Hira Sajjad Talpur, Di Wu, Farhan Anwar Khan, Dinesh Bhattarai, Zia-Ur Rehman, F. Farmanullah, Li-Jun Huo

**Affiliations:** ^1^Key Laboratory of Agricultural Animal Genetics, Breeding and Reproduction, Education Ministry of China, College of Animal Science and Technology, Huazhong Agricultural University Wuhan, China; ^2^Department of Hubei Province's Engineering Research Center in Buffalo Breeding and Products, Huazhong Agricultural University Wuhan, China; ^3^The State Key Laboratory of Agricultural Microbiology, Huazhong Agricultural University Wuhan, China

**Keywords:** nanoparticles, reproduction, animal models, human health, toxicity

## Abstract

In the last two decades, nanotechnologies demonstrated various applications in different fields, including detection, sensing, catalysis, electronics, and biomedical sciences. However, public concerns regarding the well-being of human may hinder the wide utilization of this promising innovation. Although, humans are exposed to airborne nanosized particles from an early age, exposure to such particles has risen dramatically within the last century due to anthropogenic sources of nanoparticles. The wide application of nanomaterials in industry, consumer products, and medicine has raised concerns regarding the potential toxicity of nanoparticles in humans. In this review, the effects of nanomaterials on the reproductive system in animal models are discussed. Females are particularly more vulnerable to nanoparticle toxicity, and toxicity in this population may affect reproductivity and fetal development. Moreover, various types of nanoparticles have negative impacts on male germ cells, fetal development, and the female reproductive system. These impacts are associated with nanoparticle modification, composition, concentration, route of administration, and the species of the animal. Therefore, understanding the impacts of nanoparticles on animal growth and reproduction is essential. Many studies have examined the effects of nanoparticles on primary and secondary target organs, with a concentration on the *in vivo* and *in vitro* effects of nanoparticles on the male and female reproductive systems at the clinical, cellular, and molecular levels. This review provides important information regarding organism safety and the potential hazards of nanoparticle use and supports the application of nanotechnologies by minimizing the adverse effects of nanoparticles in vulnerable populations.

## Introduction

The vast growth of nanotechnologies with all their far-reaching benefits has fostered concerns about the potential health risks of nanoparticles (NPs) (Ema et al., 2010). These products currently have extensive applications in nearly all manufacturing sectors. For example, advances in nano medicine may provide solutions for the early diagnosis of diseases and in personalized medicine with regard to treating complex diseases, such as cancer or metabolic disorders (Sanhai et al., 2008). Nanotechnologies also provide potential systems for helping to resolve societal difficulties, such as energy shortages (Le Goff et al., 2009; Li et al., 2012) and environmental pollution (Savage and Diallo, 2005). Due to their unique characteristics, NPs are widely used in biomedical and industrial applications (Lee et al., 2007; Zhang et al., 2008; Das et al., 2009; Vance et al., 2015). Currently, there are 1,814 marketed consumer products containing nanoparticles, including antibiotics, food items, textiles, sports tools, and electronic materials, and the number is increasing steadily (Chou et al., 2008; Vance et al., 2015).

**Figure 1:**
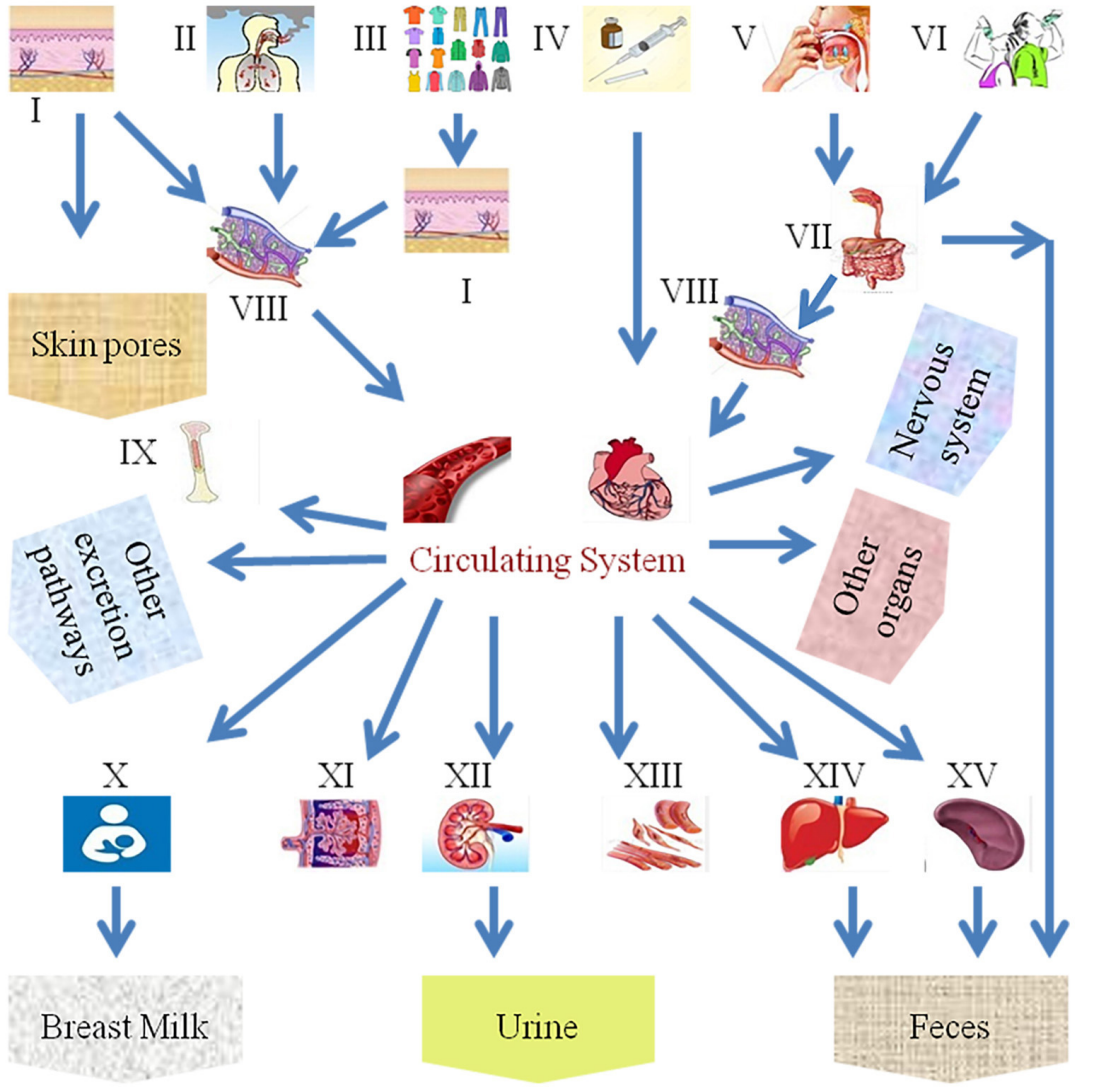
Scheme of the different exposure routes of nanoparticles in the human body. (I) Skin, (II) inhalation, (III) fabric, (VI) intravenous injection, (V) food intake, (VI) water intake, (VII) gastrointestinal tract, (VIII) lymph, (IX) bone marrow, (X) breast milk, (XI) placenta, (XII) kidney, (XIII) muscles, (XIV) liver, and (XV) spleen.

Despite the benefits of NPs, various applications of nanotechnology have exposed humans and animals to their potential toxicities. As far as the exposure of humans to NPs is concerned, they can enter the body through inhalation, ingestion, skin uptake, injection, or implantation (Figure 1; Oberdörster et al., 2005). It is also interesting to note that NP uptake could be intentional or non-intentional (Yah et al., 2012). Thus, the wide use of nanomaterials has raised concerns about the negative impact of NPs on human health, mainly on the reproductive systems of both men and women and on fetal health, particularly in view of the small size of NPs, their ease of penetration and biocompatibility and their potential ability to breach the placental barrier. Early research studies on anthropogenic NPs, for example, diesel exhaust (DE), demonstrate that, due to with daily exposure, they aggregate in and bind to human cells, disturbing normal physiological systems. Additionally, NPs are associated with different disorders in animals, including pulmonary injury, heapatotoxicity, immuno-nanotoxicity neurotoxicity, renal toxicity, and irreversible testis damage (Derfus et al., 2004; Chou et al., 2008; Lin et al., 2008; Schipper et al., 2008; Wu et al., 2011; Bartneck et al., 2012; Vance et al., 2015).

Various reports describe the pharmacological applications of NPs and the effects of environmental exposure to NPs (Chen et al., 2003; Xue et al., 2005; Kim et al., 2006). The primary objective of all the above-mentioned studies was to determine the toxicity of surface modified NPs and to analyze whether these NPs exhibited particular biological characteristics, and they were also assessed to determine if they were effective as gene or drug transfection vectors. However, their utility, in this regard, introduces another opportunity for potentially toxic effects in various organs, including the reproductive system, if the NPs are distributed throughout the body.

Fertility, reproduction, and fetal development are essential to sustain a species, highlighting the importance of the growing public awareness of the toxicity of NPs on the reproductive system. Women have only about 400 follicles that reach maturity and undergo ovulation during their lifetime, meaning that there is a limited opportunity for reproduction (Hillier, 1994; Song et al., 2009). Moreover, reproductive female organs, including the uterus and ovaries, exhibit periodic growth, and regeneration that is regulated by hormones. The hormonal control system has dynamic functions and is susceptible to the physiological stress caused by foreign particles (Warren and Perlroth, 2001; Armenti et al., 2008), and any interruption in female reproduction potentially results in fetal anomalies.

Environmental pollutants have toxic effects on reproduction and embryonic development (Anway et al., 2005; Armenti et al., 2008). Similarly, NPs present a potential threat to the susceptible female population, and their toxicity has been studied in different models of female reproductive health (Tsuchiya et al., 1996; Wang et al., 2011). Both short- and long-term toxicities in animals and humans have been documented. Additionally, several reports demonstrate the biological effects of NPs on isolated physiological systems, such as organs, biomolecules, and primary cells. Overall, such studies have raised as many questions as they have answered, and it is clear that more studies are needed to determine the mechanisms by which NPs affect particular organ systems. Moreover, NPs can distribute in different organs, and signals may be transmitted between these organ systems, affecting the entire individual. This is not limited to females. NPs can also cross the biological barriers shielding various parts of the human body, such as the blood-testes barrier and enter the testes in animal models (Araujo et al., 1999).

Many studies on the toxicity of NPs have been conducted in mice and rats, which exhibit genetic similarities to humans. Although, these are common mammalian models, their use is limited by their long developmental cycle and ethical concerns. Moreover, it is difficult to study their development *in utero*. Therefore, zebrafish have been used as a model for molecular studies, embryonic development, and developmental biology. Herein, *in vitro* and *in vivo* toxicological evaluations of NPs in animal models, such as mice, rats, and zebrafish and the impact of NPs on the male and female reproductive systems are discussed, with an emphasis on the results of the exposure to man-made NPs.

## Nanoparticle mechanisms

The development of novel nanoparticle-based technologies and products for the delivery of nanoparticles to humans is constantly expanding. The current methods for delivering nanoparticles for the treatment of human disease include oral administration, transdermal delivery, intravenous injection, or implantation (Wennerberg et al., 2011). Exposure of the human body to nanoparticles can also accidentally occur via inhalation, dermal contact, or swallowing (Figure 1). Upon entering the human circulation, nanoparticles undergo metabolism, as well as excretion and/or retention in various body compartments. Different nanoparticles have distinctly different physiochemical properties and vary in their size, shape, and surface properties, which determine how they are absorbed, distributed, metabolized, and eliminated in the human body as well as inside the cells. These processes as referred to as the absorption, distribution, metabolism, and elimination (ADME) processes and will be discussed in detail next. The adaptive evolution of mammals has led to the employment of various physiological barriers for their protection against natural and environmental hazards. One of the most important barriers of this kind in humans is the skin, which prevents absorption by the contact of nanoparticles. Despite the effectiveness of such protective mechanisms, nanoparticles smaller in size can still penetrate the skin barrier and enter human circulation (Wu et al., 2009). Upon entrance into the blood circulation, nanoparticles use the bloodstream to reach body compartments and vital organs such as the liver and spleen. In the latter organ, nanoparticles are captured by the cells of the reticuloendothelial system (RES), which is a process that is essential for the deactivation and elimination of foreign bodies (Sadauskas et al., 2007; Liu et al., 2008b). Other vital organs of the human body that nanoparticles reach include the brain (Elder et al., 2006; Wang et al., 2008), and the testis (Bai et al., 2010), or even the fetus, which are protected by their own specialized barriers. Nevertheless, even these vital organs are not fully protected, since certain nanoparticles can effectively penetrate their barriers (De Jong et al., 2008). The ability of nanoparticles to bypass/penetrate these defensive, protective barriers of the human body depends on their physical (e.g., size, shape, aspect ratio; Meng et al., 2007; Qiu et al., 2010; Ma et al., 2011) and chemical properties (e.g., aggregation, surface chemical, charge status). For example, positively charged nanoparticles can more effectively enter the cell since the cellular membrane (which consists of a double layer of phospholipids) is negatively charged. This has been also confirmed in independent experiments studying the cellular uptake of nanoparticles (e.g., polyethyleneimine-coated mesoporous silica nanoparticles), which are positively charged, demonstrating an increased uptake by cells compared to negatively charged nanoparticles (Xia et al., 2009). Thus, the increased uptake of positively charged (cationic) nanoparticles may result in increased damage of membrane phospholipids as well as increased damage to cellular compartments (e.g., the lysosomes; Xia et al., 2006). Other factors affecting the cellular uptake of nanoparticles include ligand molecules that bind to specific cell membrane receptors (Nel et al., 2009). How nanoparticles travel between different organ systems remains poorly understood. This is a process regulated by complex mechanisms and may include the translocation into the systemic blood circulation via the lymphatic vessels, as in the case of dermal or gastrointestinal absorption (Jani et al., 1990; Kim et al., 2004). In the case of inhalation, the possible routes that the nanoparticles can be transferred to include the blood (systemic) circulation, the lymphatic vessels, the gastrointestinal tract, and the central and/or peripheral nervous system (Oberdörster et al., 2005). In particular cases, such as in the case of *Caenorhabditis elegans*, ingested quantum dots can also reach the reproductive system (Qu et al., 2011), which is a process that is mainly regulated by the properties of nanoparticles, such as their surface charge (Choi et al., 2010). Given that the ADME process of nanoparticles heavily depends on their physiochemical properties (Zhang et al., 2014), Table 1 summarizes the nanoparticle properties that affect their distribution in the human body as well as the possible routes of exposure, and the main findings of the published studies. Recently, accumulating evidence has elucidated the fate of nanoparticles upon entering the human body (Wang et al., 2012). For example, the metabolism of nanoparticles entering the stomach is now better understood, and the role of cellular lysosomes in their metabolism is now well-established. The entrance of nanoparticles into cells occurs mainly by endocytosis. Nevertheless, carriage by endosomes is done in an acidic environment, which can have a potent effect on certain metallic nanoparticles, e.g., silver (Arora et al., 2008), quantum dots (Gao et al., 2004; Hoshino et al., 2004), or iron oxide nanoparticles (Arbab et al., 2005; Zhu et al., 2011), and induce their dissolution. Similarly, non-metallic nanoparticles, e.g., SiO_2_ nanoparticles (Souris et al., 2010), can also be affected by the pH of the microenvironment they are carried into and undergo dissolution intracellularly. Therefore, the development of appropriate coatings to protect nanoparticles from their dissolution could increase their durability *in vivo* and minimize their toxic effects on human cells (Li et al., 2011). Experimental studies suggest that nanoparticles undergo enzymatic catalysis [e.g., single-walled carbon nanotubes (SWCNTs) and multi-walled carbon nanotubes (MWCNTs) can be degraded by horseradish peroxidase and human myeloperoxidase]. Whether this is applicable *in-vivo* is something that has to be explored (Kagan et al., 2010). The effective clearance of nanoparticles via their enzymatic degradation in the liver or lungs minimizes their deleterious toxic effects (Kagan et al., 2010). Liver cells contain several enzymatic systems that enzymatically degrade nanoparticles (Wang et al., 2012), while lung macrophages internalize large nanoparticles, such as those with a diameter above 100 nm (Zhu et al., 2009). The surface properties of nanoparticles are other important factors that determine their fate in the human body. For example, polyethylene glycol-coating of nanoparticles prevents their endocytosis by RES cells and extends their circulation in human blood (Liu et al., 2008b). Other chemical properties of nanoparticles, such as their stereoisomeric form, also determine their toxicity. For example, d-glutathione coated cadmium telluride (CdTe) quantum dots have a lower cytotoxicity compared to l-glutathione (Li et al., 2011). When entering the human body, nanoparticles can undergo passive modifications that might differentially affect their metabolism (Lundqvist et al., 2008; Walkey and Chan, 2012). One such passive modification is the adsorption of proteins on their surface, which forms a corona. This contributes to a more effective recognition by immune cells and improved clearance (Ruge et al., 2011) or conversely attenuates their aggregation and prevents them from undergoing phagocytosis (Geiser, 2010). Other modifications that the nanoparticles undergo in the human body relate to the cleavage of surface molecules in endosomes and the absorbance of other moieties. These physico-chemical properties of the nanoparticles and their ADME processes are taken advantage of during drug delivery approaches to maximize the efficiency of the therapeutic strategies (Nahire et al., 2013), while the metabolism of nanoparticles plays a main role in determining/ minimizing any toxic effects. However, in this area, our understanding is insufficient (Kagan et al., 2010). Nanoparticles can affect physiological and metabolic processes *in vivo*, due to the high electron density on their surface (Berry, 2013). For example, graphene oxide nanoparticles serve as hydrogen peroxide catalase systems (thanks to their high electron density on their surface) and transform H_2_O_2_ to OH in *C. elegans* (Zhang et al., 2012). These properties of nanoparticles have implications for the redox state of cellular systems. By interfering in redox-related reactions, nanoparticles can alter redox-sensitive pathways in cells and induce toxicity (Nel et al., 2006; Zhang et al., 2012). This means that nanoparticle use as a drug delivery system in the human body needs to be implemented with extreme caution due to potential side effects, which may be even more pronounced in disease states, such as diabetes or aging. What is important and still poorly explored is how the metabolism of nanoparticles potentiates their toxicity. For example, metallic nanoparticles do release metallic ions intracellularly upon metabolism, which may induce cellular toxicity (Derfus et al., 2004; Fukui et al., 2012). Thus, in the cases of metallic ions or quantum dots, it is difficult to assess the toxicity of nanoparticles. Improvements in their synthesis and possible chemical modifications can be useful ways to prevent ion release and the toxic effects of nanoparticles. The physicochemical properties of nanoparticles also affect their excretion from the human body. The excretion (partial or total) of nanoparticles from the human body occurs though various routes (Figure 1). For example, the hepatobiliary route is responsible for the excretion of liposomes (Alexis et al., 2008), and SWCNTs coated with polyethylene glycol are excreted primarily via feces and urine (Liu et al., 2008b). Nanoparticle size also determines their route of excretion, such that larger nanoparticles (e.g., >80 nm in diameter) that enter the liver and spleen are excreted in feces slowly (Alexis et al., 2008), while smaller ones (diameter < 10 nm) are cleared by the renal pathway (Longmire et al., 2008). Nanoparticles that are trapped by RES cells are degraded intracellularly in macrophages, while non-degradable nanoparticles stay in human tissue systems for an extended period of time (Chen et al., 2008; Zhao et al., 2011). In addition to their excretion in feces and urine, nanoparticles can be also be excreted in saliva, sweat, and breast milk (Li et al., 2010). *in vitro* studies in lower organisms, like *C. elegans*, are useful for the better understanding the ADME processes of nanoparticles. For example, the metabolic pathway that CsSeZnS and CdTe quantum dots nanoparticles undergo in *C. elegans* is significantly different from E. coli and involves the oxidation of selenium and its distribution to the intestines and reproductive system (Qu et al., 2011).

**Table 1:** Some of the main physicochemical properties of nanoparticles, as well as the exposure routes, and main findings.

**Animal model**	**Administration route and exposure time**	**Nanoparticle**	**Surface chemistry**	**Size/nm**	**Major observations**	**References**
Mouse	i.p and i.v. injection, 1, 4, and 24 h	Gold	Without surface modification	2, 40	Macrophage uptake in liver, less in spleen, small intestine, lymph nodes.	Sadauskas et al., 2007
Rat	i.v. injection, 24 h	Gold	Without surface modification	10–250	NPs of 10 nm entered testis and brain.	De Jong et al., 2008
Mouse	i.v. injection, 0.5, 2, and 24 h	MWCNTs	Carboxylated and aminated surface	20–30 × 0.5–2 mm	Accumulation in testis.	Bai et al., 2010
Mouse	i.v. injection, 0.17, 1, and 24 h	SWCNTs	Without or coated by paclitaxel (PTX) -polyethylene glycol (PEG)	1–3 × 100 (diameter × length)	Accumulation in liver and spleen, less in heart, lung, kidney, stomach, intestine, muscle	Liu et al., 2008a
Rat	Whole body inhalation, 12 days	MnO2	Without surface modification	30	Accumulation in CNS via olfactory bulb	Elder et al., 2006
Pig	Intradermal injection, < 5 min	CdTe (CdSe) core (shell) type II QDs	Oligomeric, Phosphine	10 (naked); 18.8 (coated)	Accumulation in sentinel lymph node	Kim et al., 2004
Rat	Gavage	Polystyrene microspheres	Without surface modification	50, 100, and 300	Accumulation in liver and spleen via lymph	Jani et al., 1990
Mouse	Intranasal instillation, 2, 10, 20, and 30 days	TiO2	Without surface modification	10, 25, and 60	Accumulation in brain through olfactory bulb.	Wang et al., 2008
Hairless mouse	Dorsal skin exposure 60 days	TiO2	Hydrophobic or hydrophilic surface	80, 155	Accumulation in spleen, lung, kidney, and brain	Wu et al., 2009

In addition, it is important to understand how nanoparticles differ from their bulk counterparts. For example, the dissolution processes, which involve the release of metallic cations, are greater in NPs than in bulk materials and are influenced by a number of factors, such as the reduced size, the high surface-to-mass ratio, the high radii of curvature, and the corresponding low coordinated atoms at the surface of the NPs (Casals et al., 2012). This ability of NPs to release ions in an aqueous environment can have detrimental health effects, and specifically, silver ions released by NPs can even damage the membranes of sperm (Auffan et al., 2009). Moreover, studies demonstrate a correlation between NP toxicity and the ions released from Ag-NPs and ZnO-NPs compared to their bulk counter parts (Dibrov et al., 2002; Morones et al., 2005; Franklin et al., 2007). Additional novel properties that differentiate nanomaterials from bulk materials are typically related to size. Although, the size at which the material displays the different properties compared to its bulk material depends on the material in question (Kedziora et al., 2013). When looking from a biological point of view, matching the size of NPs with the naturally occurring functional units or components of living organisms would, thus, mostly likely make them more active in biological systems then their bulk counterparts, aiding in function or causing toxicity (Buzea et al., 2007).

In summary, nanoparticles exhibit a wide variety of physico-chemical properties and ADME properties too. This has implications on their absorption, and distribution to human body organs as well as their metabolism *in vivo* and their excretion. The latter process can be either complete or partial, implying that an amount of nanoparticles can be trapped in the human body for an extended period of time, which, thus, increases their potential harmful effects on human cells. Similar to small moieties used as drugs, nanoparticles enter the human body via various routes and undergo an ADME process, which is determined by their chemical properties and their size, but may be unique and radically different to that of small molecules (Zhang et al., 2014). Further studies on the ADME processes of nanoparticles are expected to enhance our understanding about their *in vivo* effects and their potential use as drug delivery systems. Table 2 summarizes the differences in ADME between nanoparticles and small molecules.

**Table 2:** The property differences in the adsorption, distribution, metabolism, and excretion between nanoparticles and small molecules.

	**Absorption**	**Distribution**	**Metabolism**	**Excretion**
	Entrance portal	Distribution carrier Interaction with plasma Passage through intercellular gap, (e.g., tight junction glomerular filtration) Passage through cell membrane	Mediator Biological activity change Site	Major pathway Excretion efficiency
Nanoparticles	Oral, respiratory, dermal, injection, implantation, cross barrier like skin, gut wall, alveolar membrane	Blood circulation; lymph circulation Protein corona on NPs surface Cutoff size loosely applies and many leaks Endocytosis Membrane penetration and frustrated phygocytosis (for needle like NPs)	Peroxidase enzyme (e.g., myeloperoxidse); physiological microenvironment (e.g., acidic environment in endosomes) Help excretion; decrease toxicity; targeted drug delivery Mainly in intracellular endosomes of macrophages in RES organs	Urine and feces More difficult
Small molecules		Protein-drug complex Cut off size applies Diffuse through membrane pore and lipid bilayers carrier mediated transport; endocytosis	Phase I, II enzymes; physiological microenvironment (e.g., gastric acid) Increase or decrease toxicity Mainly in liver	Urine and feces Easier

## Nanoparticles and oxidative stress

**Figure 2:**
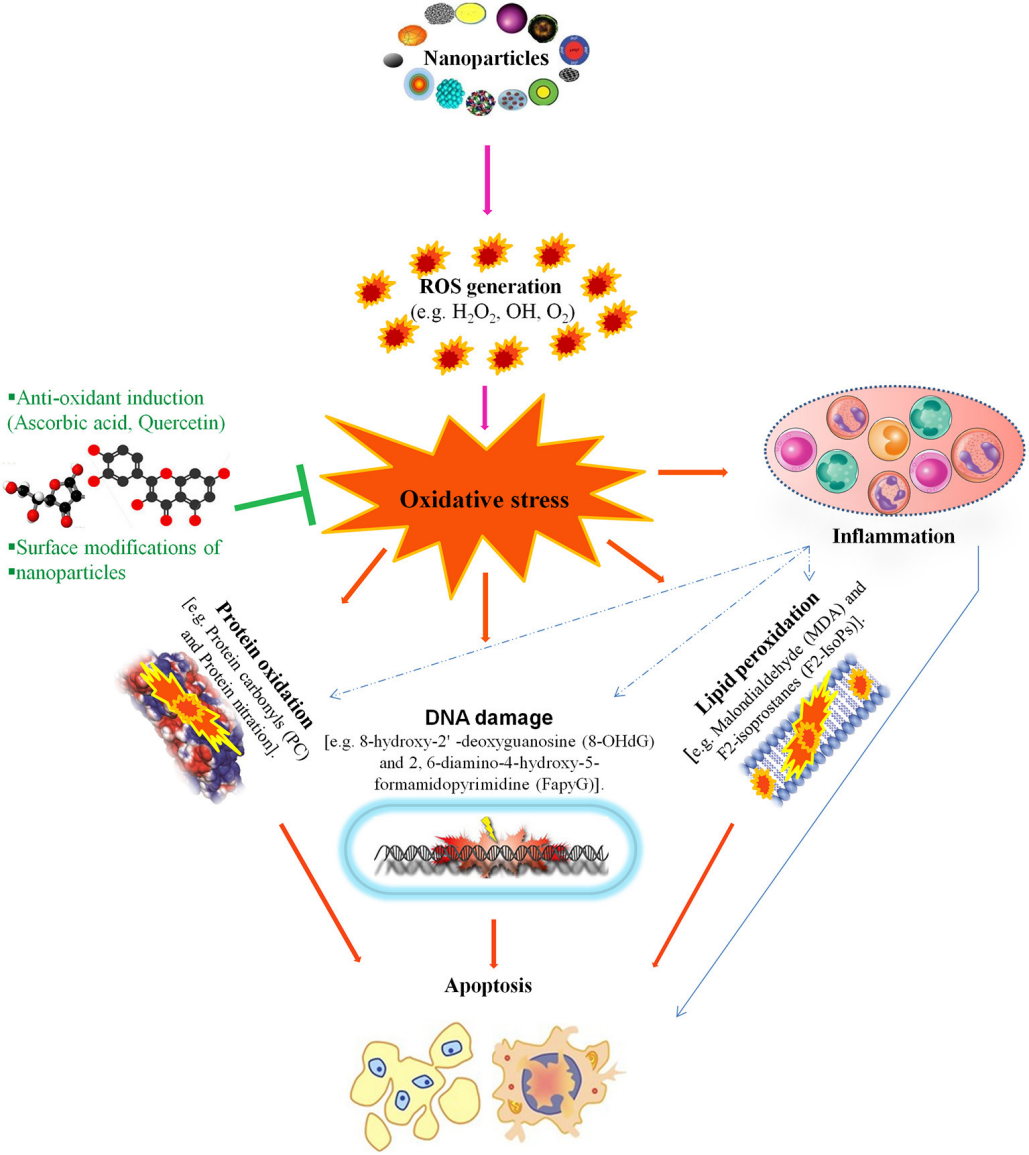
This figure illustrates that nanotoxicity produced by overproduction of free radicals which induced oxidative stress. Oxidative stress causes lipid peroxidation, protein oxidation and DNA damages, these all together potentiate inflammatory response by implying variety of inflammatory pathways. On the other hand, antioxidant defense encounter the production of oxidative stress and ameliorate reproductive nanotoxicity of animal models. Modified from Khanna et al. (2015).

NP-mediated toxicity is a major focus of many studies regarding the utilization of NPs but it is still not well understood. *in vivo* and *in vitro* studies reveal that NPs induce toxicity by increasing intracellular reactive oxygen species (ROS) levels and/or the levels of pro-inflammatory mediators. NP-induced ROS alters the homeostatic redox state of the host. NPs activate nuclear factor-kappa B (NF-κB) signaling by up-regulating the transcription of various pro-inflammatory genes, including tumor necrosis factor-α and interleukins (IL)-1, IL-6, and IL-8, which leads to oxidative stress, followed by severe DNA damage and apoptosis (Figure 2; Khanna et al., 2015).

One example of this type of toxicity occurs with nanosilver particles, which enter the cell via diffusion or endocytosis, leading to mitochondrial dysfunction and the generation of ROS, which damages proteins and nucleic acids inside the cell and, finally, inhibits cell proliferation (McShan et al., 2014). However, the molecular mechanisms underlying nanotoxicity are not entirely understood. Although, because it is well established that oxidative stress is a key determinant of NP-induced injury, the characterization the ROS response resulting from NPs is necessary. A better physico-chemical characterization and understanding of the multiple signaling cascades activated by NP-induced ROS, will contribute to NP-induced injury studies (Manke et al., 2013). Indeed, as we described below, there is evidence that NP-induced toxicity via ROS is a major factor related to how NPs affect the reproduction system in animal models.

## Nanotoxic effects on reproduction and development

**Figure 3:**
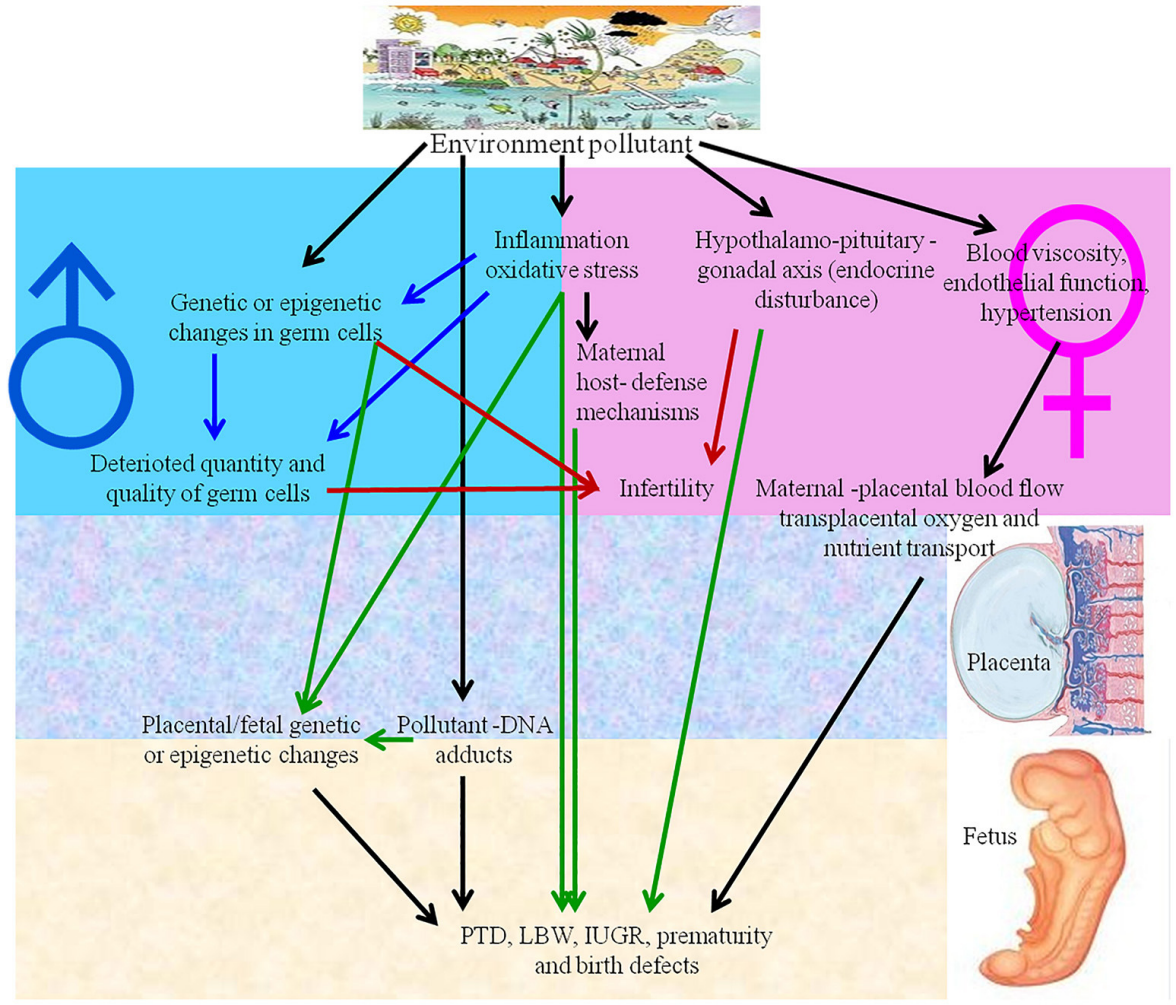
Environmental pollutants and nanoparticles have adverse effects on human reproduction. IUGR, intrauterine growth retardation; LBW, low birth weight; PTD, preterm delivery.

Reproductive toxicity refers to detrimental effects on any stage of reproduction and pregnancy in the human reproductive cycle, such as an interruption in the development of healthy embryos among child bearing age females (Adler et al., 2010). The side effects that impact the offspring at any stage of life as a consequence of parental exposure are characterized as developmental toxicity (Rogers and Kavlock, 1998). The negative effects of numerous natural contaminants, such as pollutants, on the development and reproduction of humans and animals (Slama et al., 2008), are shown in Figure 3. NPs also have negative effects on reproductive organ function, physiological structure, germ cells and fertility, as discussed below.

To study the effect of toxic NPs on biological systems, researchers have utilized numerous *in vitro* toxicity models. However, the problem with these assays is that NPs interfere with either the detection system or the assay materials, leading to conflicting or inconsistent results and thus, toxicological models by focusing more on *in vivo* studies (Bahadar et al., 2016). For the purpose of this review, we focused on the animal models that have been used to study various aspects of NP-induced toxicity and its affect on reproduction. Past research reveals the most frequently utilized animal models include rats and mice, but rabbit and *C. elegans* have also been a focus of these studies (Bahadar et al., 2016; Kong et al., 2016). While animal models reduce the assay interference problems experienced with *in vitro* models and provide a better study model, as described later on, there is still a need to improve the manner in which animal studies are conducted, as the precise mechanism of NP-induced reproductive toxicity is still lacking.

## Nanotoxicity and female reproduction

### Female reproductive organs and nanoparticles

**Figure 4:**
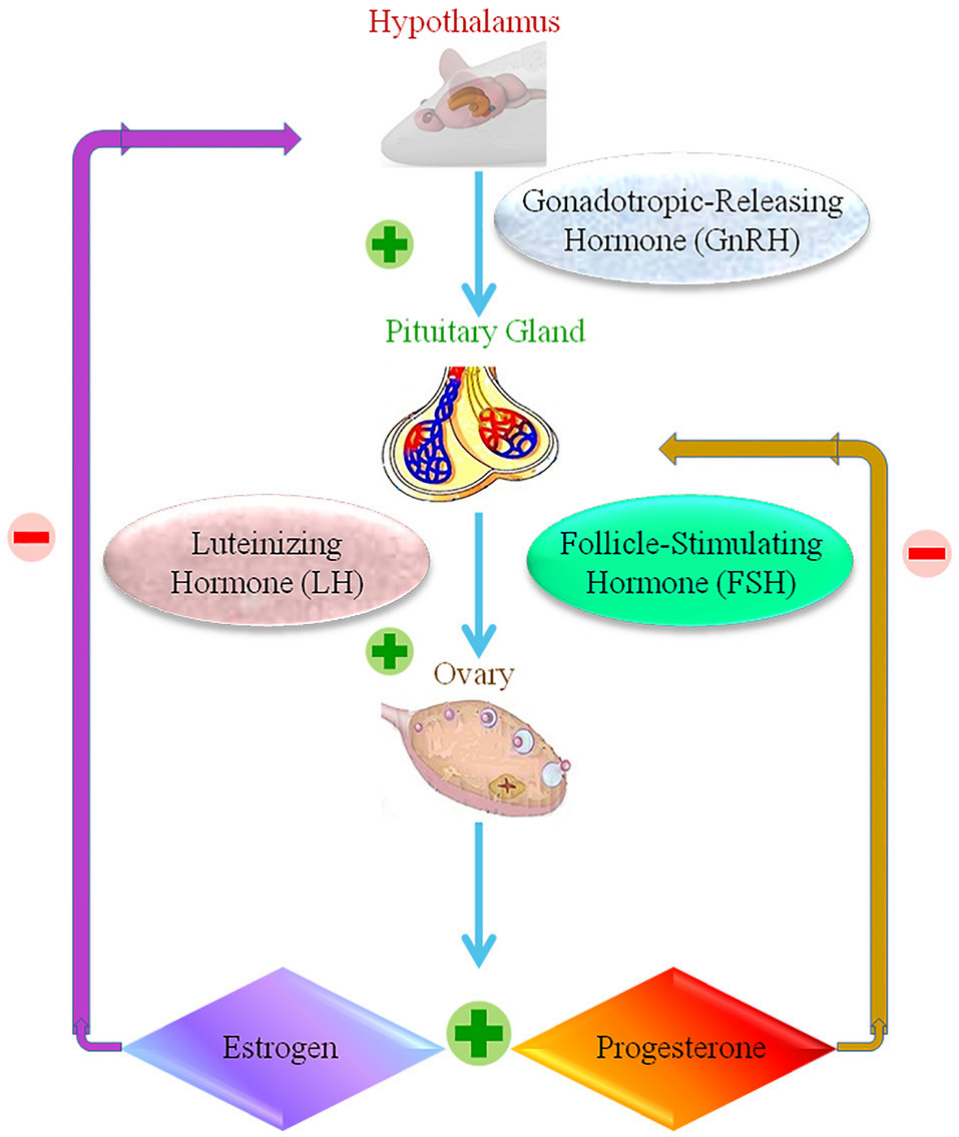
The female reproductive normal operation, showing positive and negative response within the hypothalamic-pituitary ovarian axis. 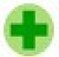 Stands for positive response. 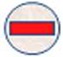 Stands for negative response.

The reproductive structure of female animals involves the reproductive organs, including the ovary, oviducts, uterus, vagina, external genitalia, and the hypothalamic–pituitary–gonadal axis. Multiple feedback mechanisms between different components of this axis are required to carry out the normal functions of reproduction (Apter, 1997; Figure 4). The accumulation of NPs is observed in female reproductive organs in animal studies, but information on whether this results in toxicity is lacking. Studies differ in terms of the type, size, dosage and length of exposure of the NP, technique of administration, and animal model used. These studies tend to focus the impact on the uterus and ovaries.

There is some evidence to suggest that smaller sized NPs are more likely to accumulate in the uterus than larger NPs. Similarly, the solubility factor also plays a major role in nanotoxicity. One recent study in rats, indicated that 1.4 nm gold NPs (AuNPs), administered intravenously at 5 μg per rat, accumulated at the uterine wall at concentrations that were two levels of magnitude higher than for 18 nm (3 μg/rat) or 80 nm (27 μg/rat) NPs (Semmler-Behnke et al., 2014). While the detected levels of AuNPs were higher in pregnant versus non-pregnant uteri, and this was proportional to the increased size and weight of the pregnant uterus. Similarly, iron oxide magnetic NPs (IOMNs) of 10 nm were recently shown to enter the mouse uterus more readily than larger NPs of 20, 30, and 40 nm (Yang et al., 2015). Information on whether these or other types of NPs cause toxicity in the uterus is lacking, However one recent study demonstrated sex-differences in the effects of gold NPs on mice livers and kidneys, having no toxicity on the reproductive system induced by 4.4 and 22.5, 29.3, or 36.1 nm particles (4,000 μg/kg), when administered by an intraperitoneal injection to either male or female mice (Chen et al., 2013).

The nature of the NP is undoubtedly of significance in terms of its toxicity to female reproductive organs. For example, cadmium, which is also known as metalloestrogen, has an impact at several levels of female reproduction. Inhalation of cadmium oxide (CdO) NPs of either 11 or 15.3 nm diameter, in one study, resulted in the dose-independent accumulation of Cd in the uterus (Blum et al., 2012). However, a daily exposure to a higher dose of CdO NPs (230 μg/m^3^) resulted in an increasing uterine weight in pregnant mice when compared to a lower dose (100 μg/m^3^) or no CdO. This higher dose of CdO NPs was delivered at ~1 μg CdO per mouse per day, whereas at the current highest allowed levels for occupational exposure, a worker in the cadmium industry might inhale 14 μg/day. This would be a considerably lower concentration, given the relative weights of humans and mice. Nevertheless, the readiness with which NPs can be distributed to secondary organs, including the uterus makes the results of studies like these worth considering when addressing safe practice in industry and any potential medical applications (Blum et al., 2012). In particular, great caution should be exercised in exposing vulnerable populations such as pregnant women to potentially toxic NPs, including CdO NPs.

In terms of other female reproductive organs, there is evidence of the accumulation of NPs in these areas. However, there is a lack of overall clarity on their potential toxicity. In the ovaries, for example, a short-term oral administration of titanium dioxide (TiO_2_) nanoparticles at 0, 1, or 2 mg/kg body weight per day in rats, results in increases in the total Ti tissue levels without general toxicity (Tassinari et al., 2014). Similarly, the acute treatment of adult mice via a single oral gavage of a high concentration (5 g/kg body weight) of 25 and 80 nm TiO_2_ NPs, results in no evidence of abnormal pathological changes in the ovaries over a 2 week period (Wang et al., 2007). In contrast a, long-term intragastric treatment, over 90 days, with a TiO_2_ nanoparticle at a lower concentration of 10 mg/kg results in ovarian damage in adult mice and is accompanied by alterations in the expression of genes associated with estrogen and progesterone synthesis and metabolism (Gao et al., 2012). An *in vitro* study on prenatal follicles dissected from rat ovaries also suggests potential ovarian toxicity with high TiO_2_ nanoparticle doses (Juan et al., 2009). In this study, 25 nm TiO_2_ nanoparticles dose-dependently reduced follicle development and oocyte maturation, at rather high concentrations >25 μg/ml (Juan et al., 2009). The *in vitro* and *in vivo* effects of exposure to TiO_2_ nanoparticles in the ovaries of rats and mice is documented in three different studies (Wang et al., 2007; Juan et al., 2009; Gao et al., 2012). It is clear that studies are needed to definitively distinguish between low and high TiO_2_ dosage and acute and long-term exposure, and to apply the information obtained for assessing safer levels of exposure for humans and potential unintended effects on the ovaries, for example, the use of TiO_2_ in sunscreens, cosmetics and prosthetic implants, and its potential use in the treatment of acne and other dermatological conditions should be assessed (Wiesenthal et al., 2011; Shi et al., 2013).

AuNPs can also target the ovaries, resulting in their development as potential anticancer drug carriers in ovarian cancer (Kafshdooz et al., 2016). Nickel NPs, however, when administered to adult rats, decrease ovarian weight coefficients, increase apoptosis and the infiltration of eosinophils and inflammatory cells into ovaries, and induce vascular dilation and congestion (Kong et al., 2014). The NP size in this study was relatively large, with an average diameter of 90 nm, while the dosing at 15 or 45 mg/kg body weight was also relatively high (Kong et al., 2014). Again, therefore, there is an urgent need to address this question of likely exposure and the relative impact of different types and sizes of nanoparticles, particularly in the context of more vulnerable populations, such as pregnant women and their fetuses.

NPs are also implicated in other aspects of female reproduction beyond the physical structures, with potential implications for fertility and development of healthy pregnancies. These include alterations of ovarian gene expression, such as for genes associated with many pathways, including steroidogenesis, apoptosis, and the acute inflammatory response.

## Ovarian gene expression and function and steroidogenesis

In females, the main female sex hormones are estrogen and progesterone, which, in humans, are mainly synthesized in the ovaries or in the placenta during pregnancy. There is some evidence to suggest that different NPs can alter the expression of genes encoding proteins involved in steroidogenesis, including ovarian genes crucial to the synthesis of estrogen and/or progesterone. For example, as mentioned above, long-term intragastric treatment of adult mice with TiO_2_ nanoparticles (5–6 nm) at 10 mg/kg, as well as causing ovarian damage, also resulted in alterations in the expression of genes in the pathways of estrogen and progesterone synthesis and metabolism, including cytochrome P450 17A1 (Cyp17a1) and aldo-keto reductase family 1, member C18 (Akr1c18) (Gao et al., 2012). Cyp17a1 is an enzyme that converts both pregnenolone and progesterone to their 17-hydroxy forms and further converts these forms to DHEA and androstenedione, respectively, thus placing it at the center of the progesterone metabolism and estrogen biosynthesis pathways. The expression of the Cyp17a1 gene, which encodes cytochrome P450 17A1, is increased upon long-term TiO_2_ NP exposure, feeding into an increase in estradiol (Gao et al., 2012). The expression of the Akrc18 gene, which encodes aldo-keto reductase family 1 member C18 (20-alpha-hydroxysteroid dehydrogenase; 20-alpha-HSD), is also increased. This is consistent with observed reductions in progesterone levels in TiO_2_ NP-treated mice, as 20-alpha-HSD is associated with progesterone metabolism (Gao et al., 2012). In this study, it was also demonstrated that long-term, high dosage TiO_2_ nanoparticle treatment resulted in an alteration of the expression of apoptosis-related genes, as well as genes associated with inflammatory and immune responses, cell proliferation, ion transport, and oxidative stress (Gao et al., 2012). Thus, changes in sex steroid levels, ovarian oxidative stress and inflammation, and increased apoptosis in the ovaries may all contribute to the ovarian damage, decreased fertility and decreased pregnancy rate observed in this study, in response to TiO_2_ nanoparticles over the long-term (Gao et al., 2012).

The impact of excessive TiO_2_ on inflammation and apoptosis-associated genes is reminiscent of a recent *in vitro* and *in vivo* mouse study using high levels of silver NPs (AgNPs; Kim et al., 2004). An *in vitro* treatment of mouse ovarian follicle granulosa cells resulted in increased mitochondrial-mediated apoptosis, while the *in vivo* expression of pro-inflammatory cytokines was increased, along with the loss of germ cells (Han et al., 2016). Thus, it appears that excessive exposure to either TiO_2_ or Ag NPs has potentially catastrophic consequences for the induction of apoptosis and/or acute inflammatory responses in the ovaries. What remains unclear is, how likely it is that human females would be exposed to such high levels at average day-to-day exposure levels. These findings are perhaps more likely to be relevant in the context of workers within industries or research projects associated with TiO_2_ or Ag NPs, or to those subject to potential therapeutic uses of these nanoparticles.

In contrast to long-term intragastric TiO_2_-induced increases in estradiol, long-term daily exposure of mice to inhaled CdO NPs (230 μg/m^3^) resulted in an ~50% reduced level of 17β-estradiol and increased mRNA for the estrogen receptors Erα and ERβ in the uterus but not in the ovaries and was associated with increased uterine weight in pregnant mice. The capacity of Cd to act as an estrogen mimetic may be of relevance in this context. Cd may act as an ER agonist and interfere with implantation. The reduced levels of implantation observed among mice treated with 230 μg/m^3^ CdO NPs is consistent with this (Blum et al., 2012).

Other studies have also addressed the potential contributions of different types of NPs to dysfunctions in female steroidogenesis. Diesel exhaust (DE) particles, for example, contain NPs, which may be implicated in reproductive toxicity. One study showed that NP-rich diesel exhaust (NP-DE) administered to pregnant rats from days 1–19 of gestation reduced the mRNA expression of the enzymes cytochrome P450 side-chain cleavage enzyme and 3β-hydroxysteroid dehydrogenase, which are key to the generation of progesterone from cholesterol during luteal steroidogenesis in rats, and also the luteinizing hormone (LH) receptor (Chun-Mei et al., 2013). However, filtered DE (F-DE) had comparable effects on these parameters, as well as inducing increased levels of estradiol-17β in serum, suggesting that NPs may not be major contributors to DE-induced steroidogenesis dysfunction in the rat (Chun-Mei et al., 2013).

Thus, the nature of the NP, the dose received, and, potentially, the route of entry can result in contrasting long-term effects. Although, the overall consequences may still be reproductive toxicity. The dose and duration of the exposure also impacts the eventual distribution of the NPs to the different organs and how far-reaching their effects might be. Further consequences can arise in pregnant females if the NPs make their way across the placenta, with potentially toxic consequences for the developing fetus.

## Transplacental transfer

**Figure 5:**
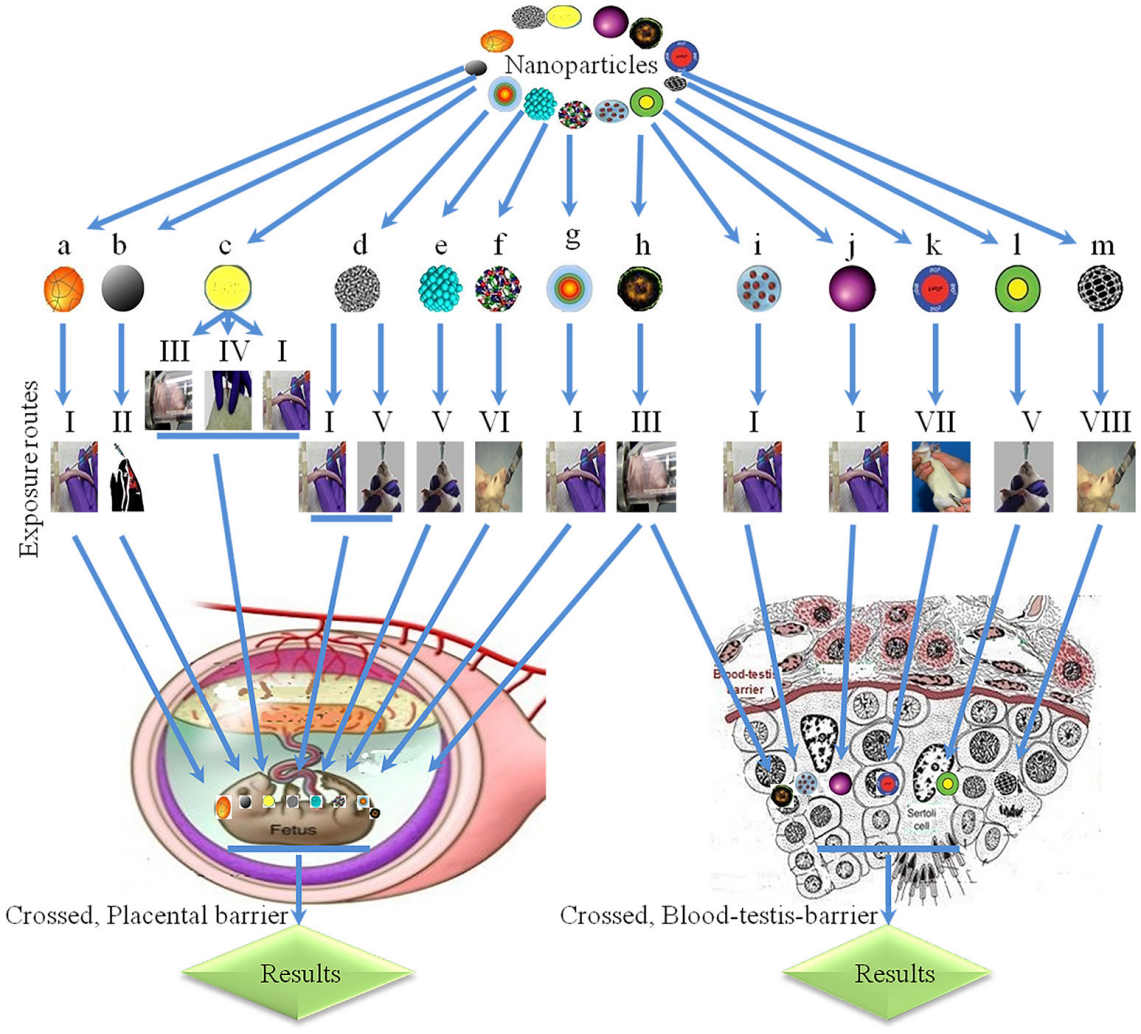
Physiological barriers and transplacental penetration of nanoparticles. **a**, ^198^Gold-nanoparticles; **b**, black carbons; **c**, titanium oxide; **d**, single-walled carbon nanotubes; **e**, platinum, **f**, multi-walled carbon nanotubes; **g**, cadmium telluride/cadmium sulfide quantum dots; **h**, diesel exhaust; **i**, sodium chloride-modified silica nanoparticles; **j**, silicon dioxide; **k**, silica-coated magnetite nanoparticles (rhodamine B isothiocyanate); **l**, metal-free polymethyl methacrylate; **m**, carbon; I, intravenous; II, intranasal; III, inhalation; IV, subcutaneous; V, oral exposure; VI, by gavage; VII, intraperitoneal; VIII, intragastric. 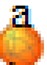
Enter fetus (Semmler-Behnke et al., 2007). 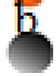
Collagen in offspring (Takahashi and Matsuoka, 1981), interrupt male reproductive system and decrease DSP (Kubo-Irie et al., 2011). 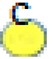
Toxic effects in embryo (Wang et al., 2007), irregular expression of genes in offspring livers (Takahashi et al., 2010), inhibition in female offspring (Fujimoto et al., 2005). 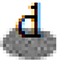
Skeletal defects and phenotypic imperfections (Snyder et al., 2015), different morphological abnormalities (Sugamata et al., 2006). 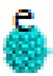
Increased mortality during lactation period and decrease growth of pups (Meng et al., 2010). 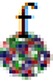
Crossed placental barrier (Jackson et al., 2011). 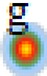
Toxic effects in embryo (Mattison et al., 1990). 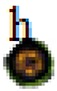
Oligospermia (Hougaard et al., 2010). Hypersensitivity in neonates and inflammation in offspring (Jackson et al., 2013), interrupt development of offspring (Kyjovska et al., 2013), disruption of sertoli and spermatozoa cells, decreased daily sperm production in offspring (Pietroiusti et al., 2011), reduces size of vesicular and prostate gland, decreased concentrations of different hormones and loss of sperm (Hamada et al., 2003). 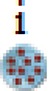
Crossed blood-testis barrier (Philbrook et al., 2011). 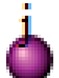
Crossed blood-testis barrier (Yoshida et al., 2009). 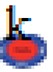
Crossed blood-testis barrier (Bai et al., 2010). 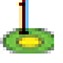
Crossed blood-testis barrier (Kashiwada, 2006). 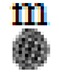
Oligospermia (Kubo-Irie et al., 2011).

The placenta produces hormones that regulate the maternal/fetal exchange and maintain gestation and embryonic growth, as well as protect the fetus from potentially harmful agents. However, the placental barrier is, out of necessity, permissive to the passage of, for example, nutrients, antibodies, and hormones, and is not completely impervious to all toxic agents, including environmental pollutants, secondary cigarette smoke, drugs, or disease-causing pathogens, with potentially deleterious consequences for the fetus (Ananth et al., 1996; Olivero et al., 1997; Perera et al., 2003; Menon et al., 2011; Robbins and Bakardjiev, 2012; Tong et al., 2013; Whidbey et al., 2013; Adebambo et al., 2015). Thus, developmental toxicity may be caused by transplacental transmission from the mother to the offspring. The small size of nanoparticles and their ready distribution to reproductive organs makes them prime candidates to breach the placental barrier. To explore this issue, different placental models have been used, including rodent and zebrafish (*Danio rerio*) embryogenesis models and perfusion models of the human placenta. Such studies confirm that NPs, such as Au, TiO_2_, SiO_2_, carbon (C), and QD NPs can readily pass through the placental barrier (Figure 5; Semmler-Behnke et al., 2007; Takeda et al., 2009; Chu et al., 2010; Sumner et al., 2010; Refuerzo et al., 2011; Yamashita et al., 2011). The effects of these NPs are size-dependent, and NPs smaller than 240 nm diameter have transplacental activity in an *ex vivo* human placental perfusion model (Wick et al., 2010). Therefore, the placental penetration ability of NPs may rely on their type, composition, and size. However, as with studies on reproductive organs and gene expression, there is wide variability in the dose and duration of NP administration. For example, different routes of administration make it difficult to directly compare between studies.

A study by Yamashita et al. (2011) was designed to assess the transplacental transfer of silica (Si) (70 nm) and TiO_2_ NPs (35 nm) following an intravenous injection into the tail vein of pregnant mice for 2 consecutive days. The results of the study indicated that there was transfer and attachment of the particles to the placental trophoblasts. (Yamashita et al., 2011) This resulted in a 20–30% reduction in uterine weight, increased fetal resorption rate, and smaller fetuses at gestational days 16 and 17, all resulting from placental dysfunction. Notably, fullerenes (0.7 nm) and Si (300 and 1,000 nm) NPs did not enter the placental trophoblast or cause adverse outcomes. Additionally, such complications were abolished by surface modification of the Si NPs with amine or carboxyl groups. However, the NP doses used in this study were very high, at 800 μg/mouse, which may represent an effective “overload.” Such doses are representative of those used in preclinical studies in animal models to test silica particle drug delivery effectiveness, but would far exceed what would be likely to result, for example, from the topical application of TiO2 NP-containing sunscreens or cosmetics (Yamashita et al., 2011). The lower doses used in this study (200 or 400 μg/mouse) did not exert the fetal consequences observed at the 800 μg/mouse dose. Another more recent study aimed to determin the mechanisms used by the NPs to cross the placenta, employing the AuNPs at much lower concentrations (Semmler-Behnke et al., 2014). The NPs, at sizes of 1.4 nm (5 μg/rat), 18 nm (3 μg/rat), and 80 nm (27 μg/rat), were intravenously injected into pregnant and non-pregnant rats. While all three sizes of NPs entered the placenta and amniotic fluid, the smallest NPs were present at orders of magnitude higher concentrations than the larger particles, which was also the case for the uterine wall. The translocation appeared to occur via the transtrophoblastic channels and/or by transcellular processes. However, no NPs were observed in the fetuses. Another study using 20 and 50 nm AuNPs in mice similarly reported the presence of Au in the placenta but not in fetal organs, with no evidence of toxicity to the placenta or fetus (Rattanapinyopituk et al., 2014). This study did report increased endocytic vesicles in syncytiotrophoblasts and fetal endothelial cells in the maternal-fetal barrier, and proposed a role for clathrin- and caveolin-mediated endocytosis in the AuNPs crossing of the placenta (Rattanapinyopituk et al., 2014). While a different type of NP was used in the studies by Semmler-Behnke et al. (2014) and Rattanapinyopituk et al. (2014) compared to that of Yamashita et al. (2011) the difference in the distribution of NPs to the fetus may also have been due to the very high concentrations of NPs used in the Yamashita study. This might result in open pathways that would not normally be functional, a phenomenon that was previously demonstrated in lung tissue when NP overload occurs (Yamashita et al., 2011; Semmler-Behnke et al., 2014).

The preferential tendency of smaller versus larger NPs to cross the placenta to the fetus has, in fact, been recognized in animal models for some time. In one study, 20 μg of radioactively labeled Au-colloid particles were injected into a Wistar rat tail vein, and 5 nm NPs were reported in fetuses at 19 gestational days at a higher transfer rate than the 30 nm NPs (Semmler-Behnke et al., 2014). NPs of both size were found at 100- to 300-fold higher levels in the placenta and fetal membrane as opposed to the fetus itself, suggesting that the NPs may have crossed the yolk sac and chorioallantoic placenta to penetrate the fetus (Takahashi and Matsuoka, 1981). However, in another similar study in mice, no transfer was observed 1, 4, and 24 h after intraperitoneal or intravenous injections of AuNPs (2 and 40 nm) in pregnant C57BL/6 mice (Sadauskas et al., 2007). Inconsistencies in the findings between studies add to the overall lack of consensus in the field. The utilization of different NP preparation protocols in various laboratories, for example, may contribute to these inconsistencies.

Other types of NPs also follow the pattern of more transfer of smaller particles. For example, fluorescent QDs have potential for use in diagnostic and biomedical imaging applications. However, in a study in pregnant mice, Cd telluride QDs (CdTe/CdS QDs) were dose-dependently transferred across the placenta to fetuses, with smaller QDs being more easily transferred than larger QDs (Chu et al., 2010). The transfer was reduced by coating with an inorganic silica shell or with polyethylene glycol (PEG), but some transfer still occurred. Such studies are important, as they indicate how the clinical utility of some NPs would be potentially limited in pregnancy.

A confounding issue with studies in rat or mouse placenta is the difficulty of extrapolating the results to humans, as the human placental physiology and anatomy are unique (Wick et al., 2010). For example, the rat and mouse placenta have three trophoblastic layers on the maternal side compared to the one layer in the human placenta (Wick et al., 2010; Rattanapinyopituk et al., 2014). This suggests that higher concentrations of NPs may be likely to penetrate the human compared to rodent placentas, with, potentially, a greater exposure to the fetus at lower NP doses. In one study, an *ex vivo* human placental perfusion model was used to attempt to assess the transfer of NPs across the human placenta specifically (Wick et al., 2010). Fluorescent polystyrene beads of diameters 50, 80, 240, and 500 nm were perfused at a concentration of 25 μg/ml, which is a rather high concentration that might be expected to resemble concentrations used in clinical applications, such as magnetic fluid hyperthermia, as opposed to regular environmental exposure (Thiesen and Jordan, 2008; Wick et al., 2010). A size-dependent translocation of the NPs across the placenta was observed, with NPs larger than 240 nm barely passed the placental barrier. The NP translocation had no observable impact on placental viability or structure. The critical nature of the NP composition is highlighted by comparing the results of this study to those of a similar study carried out using PEG-coated AuNPs in an *ex vivo* human placental perfusion model (Myllynen et al., 2008). In this case, 10 or 30 nm NPs did not cross the placenta into the fetal circulation. The limitations of *ex vivo* human placental perfusion models should be noted, i.e., that they are limited to a time period of a few hours due to subsequent tissue degradation, and therefore, they cannot be used to determine what happens over a period of chronic NP exposure. Furthermore, they give a picture of the placenta only at the final pregnancy stage, when the barrier between the maternal and fetal compartments has thinned considerably. Earlier in the pregnancy, the perfusion rates may be lower (Wick et al., 2010). Nevertheless, the results of these types of studies highlight the necessity of individually considering different types of NPs and their capacity to cross the placenta at different gestational stages. Indeed, returning to animal studies, the findings from one study in which AuNPs were injected into the tail vein of mice at concentrations between 0.9 and 7.2 mg/kg body weight suggest that the extent of fetal exposure is dependent on the gestational stage, including the stage of placental maturation (Yang et al., 2012). In fact, it was during early gestational stages, prior to 11.5 days, that the NPs primarily accumulated in the fetal tissues, whereas the extra-embryonic tissue accumulation increased after this point. The surface composition of the NP is also revealed as crucial in this study, as both ferritin- and PEG-modified nanoparticles accumulated in both fetal and extra-embryonic tissues at a greater rate than citrate-capped nanoparticles (Yang et al., 2012).

The placenta grows after uterine implantation of the blastocyst into the maternal endometrium, and its cellular composition and its structure changes throughout pregnancy (Wick et al., 2010; Yang et al., 2012). Thus, as studies suggest, NPs may affect the placenta and fetus in an exposure time-dependent manner and the defensive capacity of the embryo against exogenous toxicants may shift. A study on murine pregnancy (Yang et al., 2012) suggests extra fetal vulnerability at the early stages of pregnancy. However, the thinning of the stromal cell layer between the syncytiotrophoblast layers facing the maternal compartment and the endothelial layer facing the fetal compartment later in human pregnancy should not be overlooked, as it may facilitate a later vulnerability of the fetus to any potentially toxic NP effects (Wick et al., 2010).

## Fetal development and nanoparticles

### General fetal nanotoxicity

There is considerable evidence to suggest the potentially toxic effects of various types of NPs on the fetus, which were described in the preceding section, with deleterious consequences for fetal development. Yamashita et al. (2011), for example, observed the accumulation of TiO_2_ and SiO_2_ nanoparticles in rat fetuses, with an associated toxicity as evidenced by an increased fetal resorption rate, and smaller fetuses at gestational days 16 and 17, at a high IV dosage of 800 μg per mouse but not at lower dosages (Yamashita et al., 2011). Other studies on similarly sized AuNPs, by contrast, did not lead to the accumulation of gold in rat fetuses or any evidence of fetal toxicity, despite evidence of nanoparticle transport across the placenta into the uterus (Rattanapinyopituk et al., 2014; Semmler-Behnke et al., 2014). In these studies, the dosages of the NPs were 100- to 1,000-fold less than the TiO_2_ and SiO_2_ dosages employed by Yamashita et al. (2011). These types of anomalies highlight the difficulties in determining how much of a threat to reproductive and fetal health different types of NPs really represent, given the inherent uncertainties in determining what levels individuals are likely to be exposed to, including the relativity vulnerable people, for example, pregnant women and their fetuses.

Nevertheless, the preponderance of NPs and their biocompatibility mean that studies suggesting the potential fetal toxicity of certain NPs cannot be ignored, especially in the context of the therapeutic use of NPs in pregnant women or potentially high occupational exposure levels. Increased fetal absorption rates observed not only with high dose TiO_2_ or SiO_2_ (Yamashita et al., 2011), but also, for example, with DE elements (Fujimoto et al., 2005). Carbon-14-labeled C60 (((14) C(U))C60), for example, when administered via tail vein injection at 200–300 mg/kg to pregnant or lactating rats, crosses the placenta or is transferred via the dam's milk, resulting in increased oxidative stress in the female pups of the exposed dams (Sumner et al., 2010; Snyder et al., 2015). Meanwhile, the embryonic undeveloped cell line ES-D3 is used to investigate the developmental toxicity of AuNPs and cobalt ferrite (CoFe2O4) *in vitro* (Meng et al., 2010). AuNPs covered with hyaluronic acid and CoFe_2_O_4_ NPs covered with silanes exhibit toxic properties.

## Fetal brain and the nervous system

In animal models, the brain and nervous system are directly affected by prenatal exposure to NPs. A subcutaneous injection of TiO_2_ NPs of a diameter <300 nm administered at 100 μg/animal at gestational days 3, 7, 10, and 14 (400 μg total), resulted in the entry of NPs into the brain of the offspring of the exposed pregnant female mice, resulting in blood vessel stenosis in the hippocampus and cerebral cortex (Takeda et al., 2009). A similar experimental set-up in which 100 μg of TiO_2_ (25–70 nm) was given to pregnant ICR mice at 6, 9, 12, 15, and 18 gestational days, giving a relatively high 500 μg total dose, resulted in increased levels of dopamine (DA) and its metabolites in the prefrontal cortex and neostriatum metabolites (Takahashi et al., 2010). Moreover, the use of the same type and size of TiO_2_ NPs on days 6, 9, 12, and 15 (400 μg total) in pregnant mice revealed that in the brain tissue of the male offspring, the striatum at embryonic day (ED) 16 and post-natal day (PND) 7 and 14, the olfactory bulb at PND 2 and 14 and the cerebral cortex at PND 7, 14, and 21, were enriched in the brain gene expression changes in the regions closely associated with the DA system (Umezawa et al., 2012).

DE similarly impacts the central nervous system of the offspring of the pregnant mice, with a reduced locomotion and a decreased turnover of DA in the striatum and nucleus accumbens (Yokota et al., 2009), while apoptosis in the brain tissues is reported after 2–16 days post-coitus with a daily inhalation of DE at doses of 0.3, 1, and 3.0 particles/m^3^ in ICR mice (Sugamata et al., 2006). Meanwhile, an intratracheal administration to pregnant C57BL/6BomTac mice at gestational days 7, 10, 15, and 18, at 268 μg/animal but not at lower concentrations, induces changes in innate behavioral patterns of female offspring (Jackson et al., 2011). These studies highlight the potential vulnerability of the fetal brain to the toxicity of a variety of different types of NPs, in advance of the formation of a robust blood-brain barrier.

## Nanoparticle inhalation and the effects on offspring

NP exposure by inhalation is one of the most likely environmental exposure routes causing damage to fetal organs. The overall pathological aspects associated with these deleterious effects on the offspring still need to be deciphered. However, it is presumed that nanoparticle inhalation is associated with molecular changes in various molecular moieties involved in the developmental processes. There are several reports describing the deleterious effects of nanoparticles on offspring in animal models (Hougaard et al., 2008, 2010, 2013; Jackson et al., 2011, 2012a,b, 2013). An elaborate review on the developmental toxicity of inhaled nanoparticles warns about the toxicity of nanoparticles and suggests that a significant amount of groundwork is warranted for testing a strategy to be established on a sound scientific basis (Hougaard et al., 2015). Intriguingly, in an open field test, female offspring that were prenatally exposed to 268 ug Printex 90/animal displayed an altered habituation pattern (Jackson et al., 2011). Similarly, an intranasal instillation of pregnant ICR mice by carbon black (CB), an element of DE that can be absorbed through the airways, at a total dose of 2 mg/kg at gestational days 5 and 9, results in the increased expression of renal VIII collagen and decreased type I collagen mRNA expression in the kidneys of 12-week old offspring (Umezawa et al., 2011). This increase in nonfibrillar short-chain collagen was similar to what is observed in tubulointerstitial fibrosis in diabetic nephropathy. Although, intranasal instillation with a single bolus is not directly reflective of exposure by inhalation, it does suggest the potentially toxic consequences for offspring if there is exposure to carbon black at sufficiently high levels during pregnancy (Umezawa et al., 2011).

CB and TiO_2_ are actually considered immunologically “inert” components of DE in many immunotoxicity studies (Fedulov et al., 2008). However, a study on the intranasal exposure of pregnant versus non-pregnant mice to suspensions (50 μg/mouse) of DE particles or “inert” TiO_2_ nanoparticles showed that pregnancy increased inflammatory responses, including the levels of pro-inflammatory cytokines, to such NPs (Komatsu et al., 2008). Exposure to either NP suspension increased the chances of allergic responsiveness in the offspring of non-allergic mothers, with increased airway hyperresponsiveness and increased pulmonary inflammation, as indicated by eosinophilic pulmonary infiltration and increased bronchoalveolar lavage (BAL) levels of eosinophils (Komatsu et al., 2008). Meanwhile, exposure to TiO_2_ NP (1 h/day, 42 mg/m^3^) through inhalation pregnant C57BL/6 mice on gestational days 8-18 resulted in irregular hepatic gene expression of components in the retinoic acid signaling pathway in female offspring (Jackson et al., 2013). Other sex-specific changes induced by a daily inhalation of TiO_2_ nanoparticles (21 nm) coated with polyalcohol at a dose of 42 mg/m^3^ from gestational days 8–18 for 1 h/day included an enhancement of the prepulse inhibition in female offspring in pregnant C57BL/6BomTac mice (Hougaard et al., 2010).

It was already mentioned that inhaled CdTe/CdS quantum dots, at a dose of 230 μg/m^3^, released Cd ions in pregnant mice, resulting in altered endocrine functions, including reduced 17β-estradiol levels and increased uterus estrogen receptor mRNA (Blum et al., 2012). This has a deleterious effect on fetal attachment, disrupts the attached blastocysts and reduces fetal length and neonatal growth (Blum et al., 2012). However, the mediating effects of this mechanism of action are not yet demonstrated. The potentially toxic effects of NPs extend further to the reproductive function of the offspring, thereby providing a risk to generations following those directly exposed *in utero*.

## Nanotoxic effects on fetal reproductive function

Nanoparticle administration is associated with toxic effects on fetal development and a compromised fertility (Tsuchiya et al., 1996). Additionally, NPs may cause changes in embryogenesis and anomalies in the fetal reproductive system. Subcutaneous TiO_2_ NPs (<300 nm diameter at 100 μg/animal at gestational days 3, 7, 10, and 14; 400 μg total), results in the entry of NPs to testicular Leydig cells, sertoli cells, and spermatids in male offspring aged 4 days and 6 weeks old, as detected by electron microscopy (Takeda et al., 2009). It was also reported that nanoparticles, like DE particulates and TiO2, transiently suppress the proliferation of Leydig cells (Hong et al., 2016). In the male reproductive system, sertoli cells contribute to spermatogenesis. Interestingly, damage to these cells ultimately impacts sperm production. Exposing primary isolated sertoli cells from the mice to TiO2 results in apoptosis through lactate dehydrogenase release (Ritz et al., 2011). In a 6-week old mice, testicular morphology is altered and daily sperm production (DSP) is reduced (Takeda et al., 2009). In another study, along similar lines, maternal exposure to diesel exhaust particles enhances mutations in male germlines during development (Boisen et al., 2013). As far as the female murine germ cells are concerned, nanosized carbon black (Printex90) NanoTIO2 does not induce mutations in female murine germ cells (Boisen et al., 2012; Zhao et al., 2013). However, although this is good news, it still needs to be confirmed by evaluating the parallel effects on both males and females, as another study showed that injury to the ovaries associated with nano-TiO2 exposure (Di Bona et al., 2015). High doses (100 mg/kg) of intra-peritoneal positively-or negatively charged Fe_2_O_3_-NPs to pregnant mice also has consequences for the reproductive health of both female and male offspring (Tsukue et al., 2002). Both positively and negatively charged NPs induce morphological alterations of the uteri, while positively charged NPs also altered the morphology of the testes in the surviving offspring. This has far-reaching consequences, as positively-charged NPs when given after gestational day 10, cause both short-term fetal loss and also increase long-term fetal loss in second generation mating (Tsukue et al., 2002).

The reproductive development of offspring is also interrupted by DE, which contains fine and ultrafine particles (Ono et al., 2007; Kubo-Irie et al., 2011). Pre- and post-natal exposure of ICR (imprinting control region) mice to DE, at a concentration of 0.17 mg/m^3^, results in disruption of the sertoli and spermatozoa cells and decreases daily the DSP in male offspring (Li et al., 2009). However, the contribution of NPs to the overall reproductive toxicity of DE is more questionable. The administration of NP-rich DE compared to filtered DE (F-DE) to pregnant F344 rats, for example, between gestational days 1–19 results in comparable reductions of the relative weights of the seminal vesicle and prostate to body weight, and similar decreases in concentrations of different hormones, including serum levels of testosterone, progesterone, corticosterone, and follicle stimulating hormone and testicular levels of steroidogenic acute regulatory protein and 17β-hydroxysteroid dehydrogenase (Watanabe, 2005). Similarly, prenatal DE exposure from gestational day 7 to delivery, results in reduced levels of spermatids and sertoli cells, and, hence, reduces the daily sperm levels in the offspring, and the similarity of the results in low- and high-dose particulate exposures suggests that the toxicity is due to the DE gaseous element (Philbrook et al., 2011). Follicle stimulating hormone (FSH), receptors and mRNA expression, in contrast, increases in response to NP-rich DE only (Watanabe, 2005). Thus, it is important to exercise caution when distinguishing between the effects caused by NPs and those that are caused by other elements of DE.

Thus, the results obtained animal models with NPs, such as TiO_2_ and positively versus negatively-charged Fe_2_O_3_, nevertheless suggest that long-term effects on fertility and reproductive function in offspring of NP-exposed pregnant females cannot be ruled out. This adds another reason for the urgent clarification of the relative effects of different types of NPs, the importance of surface modification, and the definition of safe doses.

## Nanotoxic effects on fetal morphology and organogenesis

Nanoparticle administration is also associated with toxic effects on fetal morphological development and organogenesis at different gestational periods (Tsuchiya et al., 1996). An oral administration of TiO_2_ NPs in a high single dose of 100 or 1,000 mg/kg to pregnant dams causes a significant increase in fetal deformities and mortality (Park et al., 2010). It would be of interest to determine what the effects would be over a range of doses in order to more accurately reflect the likely range of TiO_2_ doses arising from environmental, occupational and therapeutic exposures. In other studies, the mortality of pups increased during the period of lactation and decreased growth without deformities, are detected after an oral administration of platinum (Pt) NPs at concentrations of 0.25, 0.5, and 1 mg/kg, 14 days before and 4 days after mating in ICR mice(Pietroiusti et al., 2011).

Meanwhile, the administration of SWCNTs (1–2 nm in diameter, 5–30 μm in length) at doses of 10 ng to 30 μg/mouse to pregnant CD-1 mice, 5.5 days after implantation, also results in skeletal defects, including divided cervical vertebra and a reduced formation of new bones in the sternum, fingers, and toes, and phenotypic imperfections (Lim et al., 2011). The lowest effective dose was determined at 100 ng. Fetal malformations are associated with increased ROS in both the placenta and fetus, and oxidized SWCNTs cause more fetal anomalies and are rated more embryotoxic than pristine SWCNTs (Lim et al., 2011). In contrast, the administration of MWCNTs by gavage, at doses up to 1,000 mg/kg/day into pregnant rats did not induce fetotoxic effects, in fetuses delivered by Cesarean section at gestational day 20 (Kyjovska et al., 2013). Thus, it appears that MWCNTs may have a less fetotoxic impact than SWCNTs, and particularly oxidized SWCNTs (Lim et al., 2011; Kyjovska et al., 2013). Clarity on this issue requires studies in which the effects of the different carbon nanotubes (CNTs) are compared directly within a single model.

## Nanotoxicity in the male reproductive system

### Seminiferous tubules and spermatogenesis

Exposure to nanoparticles also affects the male reproductive system, including an impact on spermatogenesis from the point where it begins in the seminiferous tubules of the testes. Furthermore, NP exposure as reduces sperm production (Boisen et al., 2013). However, the impact varies from species to species (Gao et al., 2013). Decreased sperm production is associated with several molecular changes by altering the overall expression of genes involved in spermatogenesis (Lan and Yang, 2012; Hong et al., 2015a,b). A review on nanoparticles on spermatogenesis suggests precautionary measures in nanomedicines and understanding the passage of nanoparticles through the blood-testes barrier (Yoshida et al., 2010). Issues can begin from the fetal stage if the NPs are transmitted *in utero*. For example, as previously mentioned, a subcutaneous administration of 400 μg total of TiO_2_ NPs (<300 nm diameter) to pregnant mice facilitates NP entry to Sertoli cells and spermatids into the seminiferous tubules and the adjacent Leydig cells, in male offspring aged 4 days and 6 weeks old (Takeda et al., 2009). This is accompanied by a reduced sertoli cell number, altered testicular morphology, with scattered and interrupted seminiferous tubules, and a reduced DSP (Takeda et al., 2009). Epididymal sperm motility is also altered in 6-week old male offspring. Thus, exposure of the fetus to TiO_2_ NPs impairs the development and function of the male regenerative system at the basic level of spermatogenesis. Similarly, an intratracheal administration of 200 μg of carbon NPs (14 nm) to pregnant ICR mice at gestational days 7 and 14 (400 μg total) results in histological changes in the seminiferous tubule and reduces DSP in male offspring at 5, 10, and 15 weeks old (Yoshida et al., 2009).

Changes in the seminiferous tubules and spermatogenesis can also be directly induced by exposing of adult male mice to NPs. An intratracheal administration of CB NPs (14, 56, or 95 nm) at a rather high dosage of 0.1 mg/mouse for 10 times every week, again results in histological changes in the seminiferous tubules and also elevates serum testosterone levels in response to 14 or 56 nm NPs (Bai et al., 2010). A smaller 14 nm CB has lesser antagonistic effects than the 56 nm NPs, despite the similar particle numbers, suggesting that NPs size may be a critical determinant on the impact on spermatogenesis (Bai et al., 2010). In another trial model, an intravenous injection of MWCNTs into male mice induced reversible damage in testes without influencing fertility (Kashiwada, 2006). Seminiferous epithelium thickness was reduced at day 15, and oxidative stress increased. However, ROS levels and seminiferous epithelium thickness were restored within 60–90 days. There was no alteration in the amount or quality of sperm or in hormone levels in the MWCNT-treated mice in the 90-day period, and mating of the treated animals resulted in normal pregnancy and delivery success rate, suggesting no influence on fertility. These results highlight the differential impact of different types on NPs and, in this case, the difference between different carbon-based NPs, namely CB NPs and MWCNT.

## Nanotoxic effects in the testes

The accumulation of NPs in the testes is demonstrated in some animal models, but an understanding of the bio-distribution of NPs in the testes remains limited and nanotoxic effects are difficult to categorically define based on the evidence from the available studies. An intravenous and/or intra-abdominal administration of Si NPs (coated with either sodium chloride or sodium iodide) at a dose of 0–225 mg/kg of body weight to Kunming white mice (Chen et al., 2003) results in NP accumulation in the testicles, among other organs, including in the glands and interstitial cells, 96 h after administration. However, there was no indication of pathological cell changes or increased animal mortality (Chen et al., 2003). Similarly, an intraperitoneal administration of silica-coated magnetic NPs containing rhodamine B isothiocyanate (MNPs@SiO_2_ RITC; water-soluble, 50-nm thickness) at 10, 25, 50, or 100 mg/kg in male IRC mice (6 weeks of age) for 4 weeks results in testes accumulation with no apparent toxic effect (Kim et al., 2006). Moreover, the administration of fluorescent NPs of 39.4 nm in diameter at 1 mg/L to Medaka fish eggs showed that the NPs were detected in the testes (Leclerc et al., 2015). However, again there were no pathological changes in the cell nuclei, and there were no acute NP associated toxicities or an increased mortality even at higher doses, including in the ensuing generations (Xue et al., 2005; Leclerc et al., 2015). By contrast, a recent study on an intramuscular injection of silica-gold NPs into mice showed no evidence of the 70-nm NPs presence in the testes even 45 days after administration (Ong et al., 2016).

Other types of NPs, however, may induce toxic effects upon accumulation in the testes, with consequences for male fecundity. Recent studies focused on AgNPs, which are associated with deleterious effects on male reproduction in mammalian models (Lafuente et al., 2016). Studies on AgNP ingestion by fruit flies suggest that AgNP accumulation in the testes impacts on the number of germline stem cells, which is discussed in more detail below (Lafuente et al., 2016). A sub-chronic oral exposure of polyvinyl propylene (PVP)-coated AgNPs to rats also leads to altered testicular histology and sperm morphology abnormalities (Hong et al., 2016). Exposure to TiO_2_ NPs is also associated with reproductive toxicity in male animal models (Ritz et al., 2011). Importantly, the relative solubility and penetration through tissue barriers might be an important factor in inducing toxicity. Soluble nanoparticles, like Ag-NP, might be excreted through normal routes and, thus, have relatively lesser toxicity, whereas insoluble particles and the ones with a long half-life will have issues, as far as the toxicity effects are concerned. Recent studies suggest that, in male mice, reduced fertility and apoptosis/necrosis of spermatogenic cells and Sertoli cells in response to TiO_2_ NP administration is related to increased inflammatory responses and compromised immunity in the testes, including impaired tumor associated macrophages/toll-like receptor 3 (TAM/TLR3) signaling (Ritz et al., 2011). Meanwhile, oligospermia was observed in ICR mice after an intratracheal injection of carbon nanoparticles (14 nm) at a high dose of 1 mg/mouse for 10 times every week (Bai et al., 2010).

There is, therefore, a pressing need to carry out comparative studies to definitively assess the accumulation of different NP types at different doses and with different delivery modes in the testes and whether this causes toxicity, especially in the context of occupational exposure for male workers and the potential consequences on fertility.

## Nanotoxic effects on germline and/or sperm cells

The potential toxicity of NPs in the reproductive system is also directly examined by analyzing germ cells. The outcome seems to depend on the type of NPs used. In one study, AuNPs (9 nm) were directly added to fresh semen from a normal healthy male to investigate the effects on human sperm (Wiwanitkit et al., 2009). When added at a dose of 44 μg/mL, the NPs accumulate in the sperm tails and head, causing immotility in 25% of the sperm. It is unclear whether it is likely that direct exposure to these levels of NPs would be likely, although the use of AuNPs in, for example, imaging applications results in rather high doses, which could in turn have an impact on sperm upon the distribution of the NPs throughout the body. The effects of directly mixing polyvinyl alcohol (PVA)-coated Fe_3_O_4_ NPs with bovine sperm were also examined (Ben-David Makhluf et al., 2006). While NPs enter sperm and attach to mitochondria in the tail and in the acrosome region in the head, there is no apparent impact on the sperm acrosome reaction and motility.

Spermatogonial stem cells from mice are a suitable *in vitro* model for comparing the nanotoxicity of different NPs in male reproductive studies (Braydich-Stolle et al., 2005). The C4 and 18 cells, established from type A spermatogonia and isolated from 6-day old male mouse and bovine sperm cells, were used to test the ability of magnetite- PVA-coated NPs to enter the sperm/primary cells without disturbing the ability of the sperm to undergo the acrosome reaction or remain motile (Braydich-Stolle et al., 2005). Mitochondrial function was assessed to give a read-out of NP cytotoxicity, along with cell morphology, membrane leakage, and apoptosis after treatment with digitonin to permeabilize the cell membrane and release the bound particles. Importantly, the NP toxicity depends on the particle dosage in the testes, whereas the solubility of salts had no significant positive effect. AgNPs were the most toxic. For example, the EC_50_ in the MTS mitochondrial function assay is 8.75 μg/ml, whereas molybdenum trioxide (MoO_3_) NPs are less toxic (EC_50_ 90 μg/ml). Such studies, which allow for a direct comparison of the effects of different types of NPs under the same experimental conditions, would help to clear up the confusion surrounding the relative effects of different NP types and dosages on the male reproductive system, as well as other areas of reproduction.

There are several industrial setups also releasing small particulate matter resembling nanoparticles. Air pollution with nanoparticles and their impact on human health is an emerging area to be explored in future. In a study to evaluate the impact of air particulate matters on living, laboratory mice exposed with ambient air in a polluted industrial area near steel mills manifested germ line mutations (Somers et al., 2002; Yauk et al., 2008). These findings are very alarming and warrant future studies aimed at evaluating the health of people living in the peripheries of industries.

## Potential mechanisms of reproductive and developmental toxicity due to NPs

NPs that are inhaled may be deposited in the airways and lungs, and due to their high surface reactivity, they have an inherent potential to induce inflammation and the generation of ROS at the site of deposition (Hougaard et al., 2010; Møller et al., 2010). In general, ROS generation is thought to be a major causative factor in the toxicity of NPs. ROS molecules are unstable and typically do not travel far beyond their site of formation (Wells et al., 2005). However, if there is an imbalance between oxidant and antioxidant mechanism, oxidative stress increases and induces or exacerbates NP-induced inflammation. In this case, neither the particles nor the inflammatory condition need is restricted to the lungs. The NPs can translocate, and inflammatory mediators might be released into the systemic circulation (Erdely et al., 2011). The NPs can be transported to organs related to pregnancy and fetal development and may be taken up by placental cells and interfere indirectly with fetal development by inducing oxidative stress and inflammation at that site. In particular, the placental cells contain toll like receptors (TLRs; especially, TLR-2 and TLR-4), which are involved in NP-induced inflammatory responses in the airway (Zhao et al., 2012; Koga et al., 2014). Clearly, there is evidence that inflammation and ROS generation might contribute to NP-induced toxicity in reproduction and development, but further studies are needed to better elucidate these mechanisms.

## Future studies that are still required

One of the main gaps in our understanding of how NPs are related to reproduction comes from the fact that studies are not designed consistently, and thus, it is hard to make comparisons across methods. To fill this gap, nano-toxicologists and reproductive scientists should work more closely together to design and interpret studies. By achieving optimal designs for the investigation of specific hypotheses and the understanding of certain aspects of the study, researchers can actually begin to fully answer important questions, such as whether there is a NP dose-effect. In addition, one of the most questions involves the mechanism of NP-induced lung and systemic inflammation. It is necessary to understand if inflammation is the driving force for developmental effects and whether the inflammatory response is different between the pregnant and the non-pregnant state. Furthermore, because of the fact that inflammation enhances particle transfer across the placenta (Tian et al., 2013), it might be important to know how development is affected if a NP exposure occurs in someone with an existing state of low-grade chronic inflammation, such as asthma and obesity, compared to NP exposure in a non-inflammatory state. Another gap to fill is the ability to compare studies with respect to exposure regimens and outcome assessments. This would require grouping of different NPs based on risk assessment and looking for patterns among different NPs. Finally, the dosing should be examined more thoroughly. There are many inconsistencies in the importance of the dose rate and the potential underlying differences for the induction of inflammation and the translocation of NPs across the lung and placenta.

## Conclusion

The volume of studies on the toxicity of NPs in the reproductive system of animals is increasing, but the field is effectively still in its preliminary stages. While there is evidence to suggest the entry of some NPs into both male and female reproductive organs, both directly in adult animals and *in utero*, the studies were carried out with widely varying doses and administration routes, making direct comparisons and definitive conclusions difficult. In female animals, targeting of the uterus and ovaries is shown for a variety of NPs, including, for example TiO_2_, Cd, and Au, but there is wide variability in the results of the studies in terms of the evidence of morphological effects. In males, again there is evidence to suggest that NPs accumulate in the testes. For example, while Si-based NPs appear to have few, if any, toxic effects, Ag and TiO_2_-based NPs may be more dangerous, with an impact on cells in the seminiferous tubules, immune and inflammatory reactions, and sperm motility and morphology. All of these present a potential risk to male fertility if safe exposure levels are not defined and applied, such as among men working in the NP industry. There is a need to clarify the likely exposures of humans and animals to different types of NPs and tailor animal studies to mirror doses and entry routes for environmental, occupational, therapeutic, diagnostic, and cosmetic uses. Transplacental transfer of many types of NPs, including Au, TiO_2_, SiO_2_, C, and QDs, is established in animal models, and there is evidence to suggest that, in many cases, this results in the transfer of NPs to the vulnerable fetus, with varying toxic effects on, for example, the fetal brain and nerve development and future fertility. Preclinical studies with clearly defined parameters are urgently needed to clarify the situation with respect to fetal vulnerability, as well as reproductive health in general, to different NP types, sizes, surface charges, treatments, doses, and routes of delivery and the potentially toxic effects thereof. For example, the inhalation of NPs is a likely route of exposure in both environmental and occupational conditions, and CB-, TiO_2-_, and Cd-based NPs, among others, have been shown to have fetotoxic effects when inhaled by pregnant animals. The definition of “safe” levels of NPs in terms of reproductive and fetal health is imperative as the use of these products widen. The challenge is to carry out better coordinated and defined animal pre-clinical studies and human clinical trials to allow these goals to be achieved.

## Author contributions

RB and LH were involved in the writing manuscript. RB, LH, LW, HT, ZR, and DB reviewed, summarized, and analyzed the literature to develop the conceptional outline to present the analysis, formulated the key summary point of review and drafted the manuscript and figures. DW, FK, and FF critically revised manuscript and assisted final review of figures, tables, and helped to the final preparation of manuscript.

### Conflict of interest statement

The authors declare that the research was conducted in the absence of any commercial or financial relationships that could be construed as a potential conflict of interest.
